# Intravenous hydrogen sulfide does not induce neuroprotection after aortic balloon occlusion-induced spinal cord ischemia/reperfusion injury in a human-like porcine model of ubiquitous arteriosclerosis

**DOI:** 10.1186/s40635-018-0209-y

**Published:** 2018-10-24

**Authors:** Andre Bredthauer, Karla Lehle, Angelika Scheuerle, Hubert Schelzig, Oscar McCook, Peter Radermacher, Csaba Szabo, Martin Wepler, Florian Simon

**Affiliations:** 1 Department of Anesthesiology, University Hospital Regensburg, Franz-Josef-Strauß-Allee 11, 93053 Regensburg, Germany; 2 Institute of Anesthesiological Pathophysiology and Process Engineering, University Hospital Ulm, Helmholtzstraße 8/1, 89081 Ulm, Germany; 3 Department of Cardiothoracic Surgery, University Hospital Regensburg, Franz-Josef-Strauß-Allee 11, 93053 Regensburg, Germany; 4 Institute of Pathology – Section Neuropathology, University Hospital Ulm, Albert-Einstein-Allee 23, 89081 Ulm, Germany; 5 Department of Vascular and Endovascular Surgery, Heinrich-Heine-Universität Düsseldorf, Moorenstraße 5, 40225 Düsseldorf, Germany; 6 Department of Anesthesiology, University of Texas Medical Branch, 301 University Boulevard, Galveston, TX 77555 USA

**Keywords:** Hydrogen sulfide, I/R injury, Ubiquitous arteriosclerosis, Aortic aneurysm, Neuro protection

## Abstract

**Objective:**

In rodents, intravenous sulfide protected against spinal cord ischemia/reperfusion (I/R) injury during aortic balloon occlusion. We investigated the effect of intravenous sulfide on aortic occlusion-induced porcine spinal cord I/R injury.

**Methods:**

Anesthetized and mechanically ventilated “familial hypercholesterolemia Bretoncelles Meishan” (FBM) pigs with high-fat-diet-induced hypercholesterolemia and atherosclerosis were randomized to receive either intravenous sodium sulfide 2 h (initial bolus, 0.2 mg kg body weight (bw)^−1^; infusion, 2 mg kg bw^−1^ h^−1^; *n* = 4) or vehicle (sodium chloride, *n* = 4) prior to 45 min of thoracic aortic balloon occlusion and for 8 h during reperfusion (infusion, 1 mg kg bw^−1^ h^−1^). During reperfusion, noradrenaline was titrated to maintain blood pressure at above 80% of the baseline level. Spinal cord function was assessed by motor evoked potentials (MEPs) and lower limb reflexes using a modified Tarlov score. Spinal cord tissue damage was evaluated in tissue collected at the end of experiment using hematoxylin and eosin and Nissl staining.

**Results:**

A balloon occlusion time of 45 min resulted in marked ischemic neuron damage (mean of 16% damaged motoneurons in the anterior horn of all thoracic motor neurons) in the spinal cord. In the vehicle group, only one animal recovered partial neuronal function with regain of MEPs and link motions at each time point after deflating. All other animals completely lost neuronal functions. The intravenous application of sodium sulfide did not prevent neuronal cell injury and did not confer to functional recovery.

**Conclusion:**

In a porcine model of I/R injury of the spinal cord, treatment with intravenous sodium sulfide had no protective effect in animals with a pre-existing arteriosclerosis.

## Background

During open aortic surgery, interrupting blood flow through the aorta by applying a cross-clamp is often a key step to allow for surgical repair. Consequently, ischemia is induced in parts of the body distal to the clamp site. This significant alteration in blood flow by cross-clamping induces hemodynamic changes. Upon release of the cross-clamp, the blood flow is restored, triggering an ischemia/reperfusion (I/R) injury, leading to tissue inflammation, humoral changes, and lactate acidosis that increases the risk of multi-organ failure (MOF) and therefore affects postoperative outcome [1]. The most vulnerable organs are the kidney (renal failure, incidence 4.6–5.6%) and the spinal cord (ischemia, incidence 2.7–13.2%) [2, 3].

Hydrogen sulfide (H_2_S) is an endogenously produced gaseous transmitter and neuromodulator, derived from l-cysteine in several organs, such as the brain, heart, kidney, and liver [4]. H_2_S plays many important roles in the central nervous system under physiological and pathological states, especially in secondary neuronal injury. H_2_S has been shown to protect the brain from I/R injury via maintenance of mitochondrial function, inhibiting pro-inflammatory factors, neutralizing reactive oxygen species (ROS), and reducing apoptosis [5]. In a rat model of aortic balloon occlusion for 12 h, pre-emptive intraperitoneal injection of 30 μmol kg^−1^ sodium hydrogen sulfide (NaSH) reduced spinal cord infarct area and improved hind motor function 48 h after aortic occlusion [6]. In this study, it was reported that the reduced spinal cord injury was due to enhanced neurocyte autophagy. As of today, no data on the effects of H_2_S on spinal cord injury after aortic clamping in large animals is available. The transferability of the efficacy of H_2_S-based therapies from rodent models into larger animals may be questioned, at least in part due to their large surface area/mass ratio [7–10]. Therefore, the aim of this study was to test the hypothesis whether an intravenous (i.v.) formulation of sodium sulfide (Na_2_S) would induce neuroprotective effects during porcine aortic balloon occlusion-induced spinal cord I/R injury using a human-like porcine model of ubiquitous arteriosclerosis.

## Materials and methods

### Animals

The experiments were performed in adherence with the National Institutes of Health Guidelines on the use of Laboratory Animals and after approval of the protocol by the regional council of Tübingen (Registration number 905). Eight downsized familial hypercholesterolemia Bretoncelles Meishan (FBM) pigs of either sex with a mean bodyweight of 64 kg (range 24–92 kg) were used. The pigs were housed and fed as described previously [11, 12]. Na_2_S for i.v. injection was kindly provided by Csaba Szabo (Department of Anesthesiology, University of Texas Medical Branch, Galveston, TX, USA) and synthetized using H_2_S gas as the starting material, which was bubbled through an aqueous solution of sodium hydroxide (NaOH) and saline, formulated to pH neutrality and iso-osmolarity. This solution was filtered and placed under N_2_ atmosphere [13–15].

### Procedure

The anesthetic procedure, surgical preparation, placement of catheters, and physiological measurements have been described in detail previously [12, 16]. Briefly, after induction of anesthesia (i.v., propofol, ketamine) and subsequent endotracheal intubation, anesthesia was maintained with continuous i.v. propofol (6–8 mg kg^−1^ h^−1^) and remifentanil (15–20 μg kg^−1^ h^− 1^). Pigs were mechanically ventilated [FiO_2_ 0.25–0.35, adjusted to keep arterial pO_2_ levels > 100 mmHg, tidal volume 10 mL kg^−1^, positive end-expiratory pressure (PEEP) 5 cmH_2_O, inspiratory/expiratory time ratio 1:1.5, respiratory rate 10–13 min^−1^ adjusted to maintain arterial pCO_2_ between 35 and 40 mmHg]. These ventilator settings were used because swine are particularly susceptible to atelectasis formation in dependent lung regions due to the lack of alveolar collateral ventilation [16]. Sodium heparin (200 IU h^−1^) was continuously infused for anticoagulation. Via surgical cut-downs, the catheters were placed in the A. carotis dextra for measurement of blood pressure in the upper half of the body (mean arterial pressure, MAP proximal), trans-pulmonary single indicator thermodilution-cardiac index (CI), and the intra-thoracic blood volume index (ITBVI), a well-accepted marker of cardiac preload [17], as well as in the V. jugularis dextra for measurement of central venous pressure (CVP) and drug infusion. Via femoral cut-down, catheter sheaths were introduced into the Aa. femorales sinistra and dextra for distal blood pressure recording (MAP distal) and placement of inflatable balloon catheters. Adapting a technique previously published by several authors [18, 19], one catheter was placed directly above the aortic trifurcation, and the other one directly downstream of the A. subclavia sinistra, the correct position of which was manually controlled via a left-sided thoracotomy. This approach was chosen to prevent any perfusion of the spinal cord via collateral flow distal to the proximal balloon [20], which could result from variable bifurcation of the A. radicularis magna anterior [21]. The intra-aortic balloon occlusion was used to avoid mechanical injury related to clamp placement and release per se [22]. After 30 to 45 min of occlusion time, the balloon catheters were deflated. We started with an occlusion time of 30 min, followed by 40 min in preliminary experiments to test the response of this pig strain to a spinal cord I/R injury. Aortic occlusion was then performed for 45 min because this ischemia time had resulted in moderate neuronal damage (5 to 15% of all motor neurons) in the spinal cord in previous experiments [23]. In addition, 45 min of aortic occlusion prevented both the large spinal cord infarction over several segments reported in pigs after a clamping period of 60 min or longer [24] and the fairly mild tissue damage observed after only 30 min of clamping [25, 26]. Pigs were normothermic at the beginning of preparation period, and body temperature dropped to 32 °C at the start of either sodium sulfide or sodium chloride infusion. Pigs of this particular strain are known to spontaneously develop hypothermia during anesthesia [12]. Hemodynamic data, motor evoked potentials (MEPs), and neuronal function were assessed during aortic balloon occlusion and at 1 h, 4 h, and 8 h of reperfusion.

### Application of sodium sulfide (Na_2_S)

Two hours before balloon occlusion, animals were treated with saline (vehicle group) or sodium sulfide (study group). The sodium sulfide infusion rate (initial bolus 0.2 mg kg^−1^ followed by continuous i.v. infusion of 2 mg kg^−1^ h^−1^ during the 2 h before aortic occlusion, and 1 mg kg^−1^ h^−1^ during the 8 h reperfusion) was based on previous studies [27]. During balloon occlusion time, sodium sulfide or vehicle infusion was stopped.

### Harvesting, measurements, and calculations

The animals were euthanized in deep anesthesia by giving a bolus of phenobarbital (Narkodorm Alvetra, Neumünster, Germany) and i.v. potassium chloride (20 mmol, Braun, Melsungen, Germany). Tissue samples of the lumbar and thoracic spinal cord were isolated immediately post mortem and fixed in 6% buffered paraformaldehyde, and standard 3-μm paraffin sections were stained with hematoxylin and eosin (HE). Particular attention was paid on the anterior horn (AH), because of the vulnerability due to a hypoxic damage [28]. In addition, spinal cord sections were analyzed after nuclear cresol violet staining (Nissl staining) for neuronal damage [26].

Spinal cord function was evaluated by MEPs as described previously [24, 29, 29, 30]. Three electrodes were inserted into the scalp, and one into the soft palate to apply electric impulses (Digitimer Ltd., MultiPulse Stimulator D185 mark IIa) to the motor cortex. To quantify MEPs, electrodes were inserted in the muscles of the limbs to measure neuronal potential (ExcelTech Ltd., ExlTek Neuromax 1004). After electric stimulation of the cerebral motor cortex, the neural responses of the upper and the lower limbs were recorded. Decrease of more than 75% of the MEP amplitude was accepted as an indication of ischemic spinal cord dysfunction [24, 29]. MEP signal disappeared within 5 min in all animals as a sign of sufficient aortic occlusion. MEPs were triggered directly before aortic occlusion as well as 1 h, 2 h, 4 h, and 8 h after reperfusion.

In addition, spinal cord function was clinically evaluated by observing the movements of the upper and lower limbs in response to claw clamping during temporarily reduced anesthesia. The muscular response was classified as follows: 0 = no movement, 1 = muscular movement, 2 = joint movement, 3 = normal movement; an additional score of 4 was attributed if spontaneous movement was present even without stimulation by claw clamping. The reaction was measured before clamping (measuring point, MP1) and 1 h (MP2), 2 h (MP3), 4 h (MP4), and 8 h (MP5) after reperfusion. The reaction of the upper extremities was used to demonstrate the normal response.

Heart rate, MAP proximal, MAP distal, CVP, ITBVI, and CI were recorded as hemodynamic parameters. Arterial blood samples were collected to analyze blood gases, acid-base status, electrolytes, hemoglobin content, O_2_ saturation, and glucose levels.

### Statistical analysis

All data are presented as median (interquartile range, IQR). A Mann-Whitney *U* test was performed for systemic hemodynamics, gas exchange, acid-base status, and histology. A *p* value of less than 0.05 was considered statistically significant. IBM SPSS Statistics software (Version 24.0.0.0) was used for statistical evaluation and graphical display.

## Results

### Mortality rates

All animals survived the experiments until the end of the observation period (reperfusion time, 8 h).

### Hemodynamics, gas exchange, acid-base status

Table 1 presents data on systemic hemodynamics, gas exchange, acid-base status, and electrolytes. There were no statistically significant differences between the two groups.

**Table 1:** Systemic hemodynamic, gas exchange, and acid-base status

Parameter	Group	NaCl	Na_2_S
Core temp. (°C)	Baseline	32 (31.8–32.1)	31.8 (31.5–32.0)
	4 h reperfusion	32 (31.8–32.0)	32.0 (31.9–32.1)
	8 h reperfusion	32 (31.8–32.1)	32.0 (32.0–32.0)
Hemoglobin (g L^−1^)	Baseline	83 (71–88)	82 (74–94)
	4 h reperfusion	101 (92–108)	101 (81–123)
	8 h reperfusion	98 (83–107)	102 (82–132)
Heart rate (bpm)	Baseline	72 (62–81)	74 (54–94)
	4 h reperfusion	89 (47–109)	112 (89–135)
	8 h reperfusion	82 (49–109)	106 (78–130)
CVP (mmHg)	Baseline	8 (5–10)	7 (4–10)
	4 h reperfusion	9 (7–10)	7 (5–9)
	8 h reperfusion	8 (7–10)	7 (4–9)
CI (ml kg^−1^ min^−1^)	Baseline	84.4 (64.2–102.8)	72.5 (61.9–78.3)
	4 h reperfusion	85.1 (48.6–125)	90.7 (70.3–121)
	8 h reperfusion	80.0 (48.8–108.3)	83.7 (54.5–120)
MAP (mmHg)	Baseline	82 (63–93)	77 (70–86)
	4 h reperfusion	82 (78–91)	84 (59–107)
	8 h reperfusion	84 (78–88)	81 (69–95)
ITBVI (mL m^−2^)	Baseline	636 (537–786)	629 (482–762)
	4 h reperfusion	727 (509–1173)	612 (496–805)
	8 h reperfusion	665 (587–780)	577 (509–742)
Arterial pO_2_ (mmHg)	Baseline	168 (147–189)	151 (134–161)
	4 h reperfusion	169 (136–187)	126 (92–150)
	8 h reperfusion	171 (145–187)	120 (91–138)
Arterial pCO_2_ (mmHg)	Baseline	39 (37–39)	38 (35–43)
	4 h reperfusion	36 (33–38)	42 (35–47)
	8 h reperfusion	39 (36–41)	42 (39–48)
Horowitz index (mmHg)	Baseline	584 (490–630)	604 (536–640)
	4 h reperfusion	562 (543–623)	505 (368–600)
	8 h reperfusion	569 (483–623)	483 (364–552)
Arterial pH	Baseline	7.48 (7.42–7.56)	7.52 (7.46–7.56)
	4 h reperfusion	7.43 (7.36–7.50)	7.37 (7.29–7.49)
	8 h reperfusion	7.41 (7.34–7.50)	7.32 (7.10–7.44)
Arterial base excess (mmol L^−1^)	Baseline	5.2 (0.8–10.8)	7.9 (3,2–11.5)
	4 h reperfusion	0,1 (− 4.6–3.6)	1.2 (− 4–3.2)
	8 h reperfusion	0.1 (− 3.8–4.5)	− 4,3 (− 15.3–2.4)
Na^+^ (mmol L^−1^)	Baseline	138 (133–141)	138 (137–139)
	4 h reperfusion	141 (139–143)	142 (139–142)
	8 h reperfusion	142 (141–143)	139 (129–144)
K^+^ (mmol L^−1^)	Baseline	3.4 (3.1–3.7)	3.8 (3.6–4.2)
	4 h reperfusion	3.9 (3.8–4.1)	4.1 (3.8–4.7)
	8 h reperfusion	3.9 (3.9–4.0)	3.9 (3.4–4.5)
Glucose (mg dL^−1^)	Baseline	114 (105–136)	144 (103–172)
	4 h reperfusion	169 (155–202)	185 (126–239)
	8 h reperfusion	159 (88–222)	160 (124–235)

All data are median (range); sodium chloride (vehicle, NaCl) *n* = 4 and sodium sulfide (Na_2_S) *n* = 4. *h* hours, *CVP* central venous pressure, *CI* cardiac index, *ITBV* intra-thoracic blood volume index, *MAP* mean arterial pressure

### Histology

**Fig. 1:**
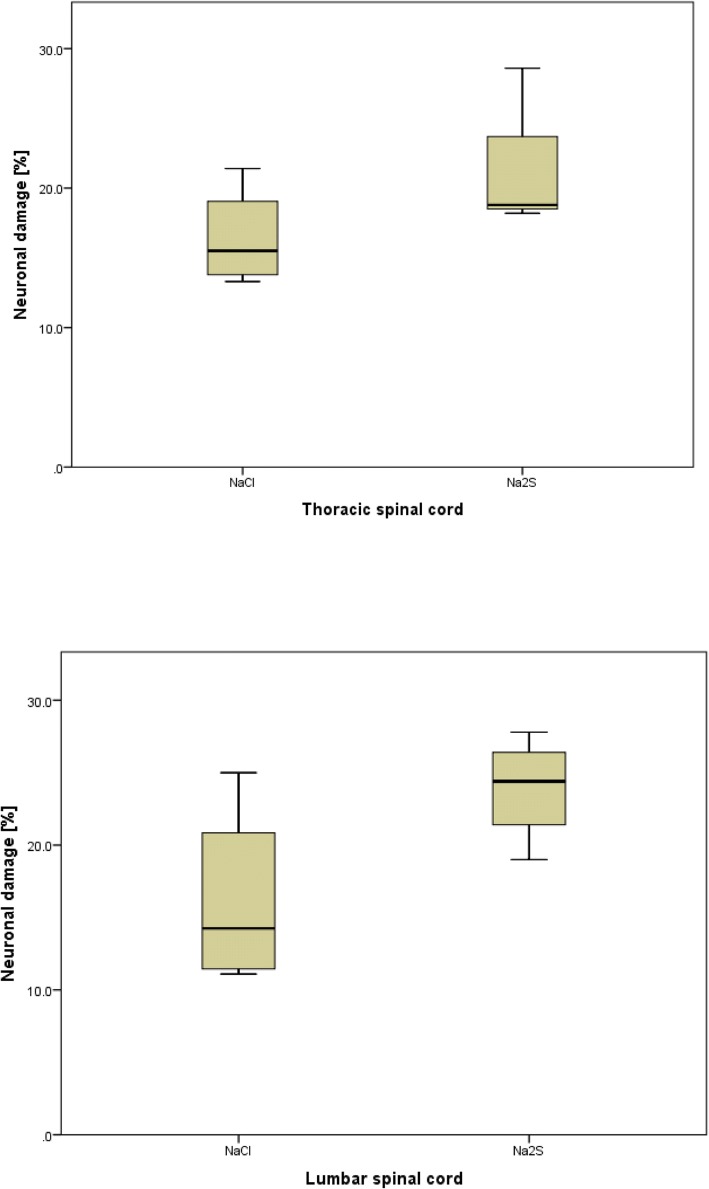
Percentage of damaged motoneurons in the spinal cord. Percentage of damaged neurons within the spinal cord at thoracic (upper graphic) and lumbar levels (bottom). Data shown as median (interquartile range). Sodium chloride (NaCl) *n* = 4, sodium sulfide (Na_2_S) *n* = 4

Animals from all groups showed the physiological structure of the spinal cord. I/R injury caused accumulations of single reactive, predominantly mononuclear round cell/glial infiltrates, atherosclerotic alterations in arterioles/arteries, and slight swelling of the perikaryon of neurons in the anterior horn of the spinal cord (Fig. 1) of all animals. Two pigs from the vehicle group developed an infarct at both levels of the spinal cord. The cross sections presented edema, eosinophilic necrosis of ganglion cells, and motor neurons. The other animals of this group were without other pathological findings. One pig of the sulfide group showed an acute infarct with hypoxic necrosis of ganglion cells, endothelial cell damage, and edema in the lumbar and thoracic spinal cord. The remaining three animals from the sodium sulfide group were without other pathological findings.

**Fig. 2:**
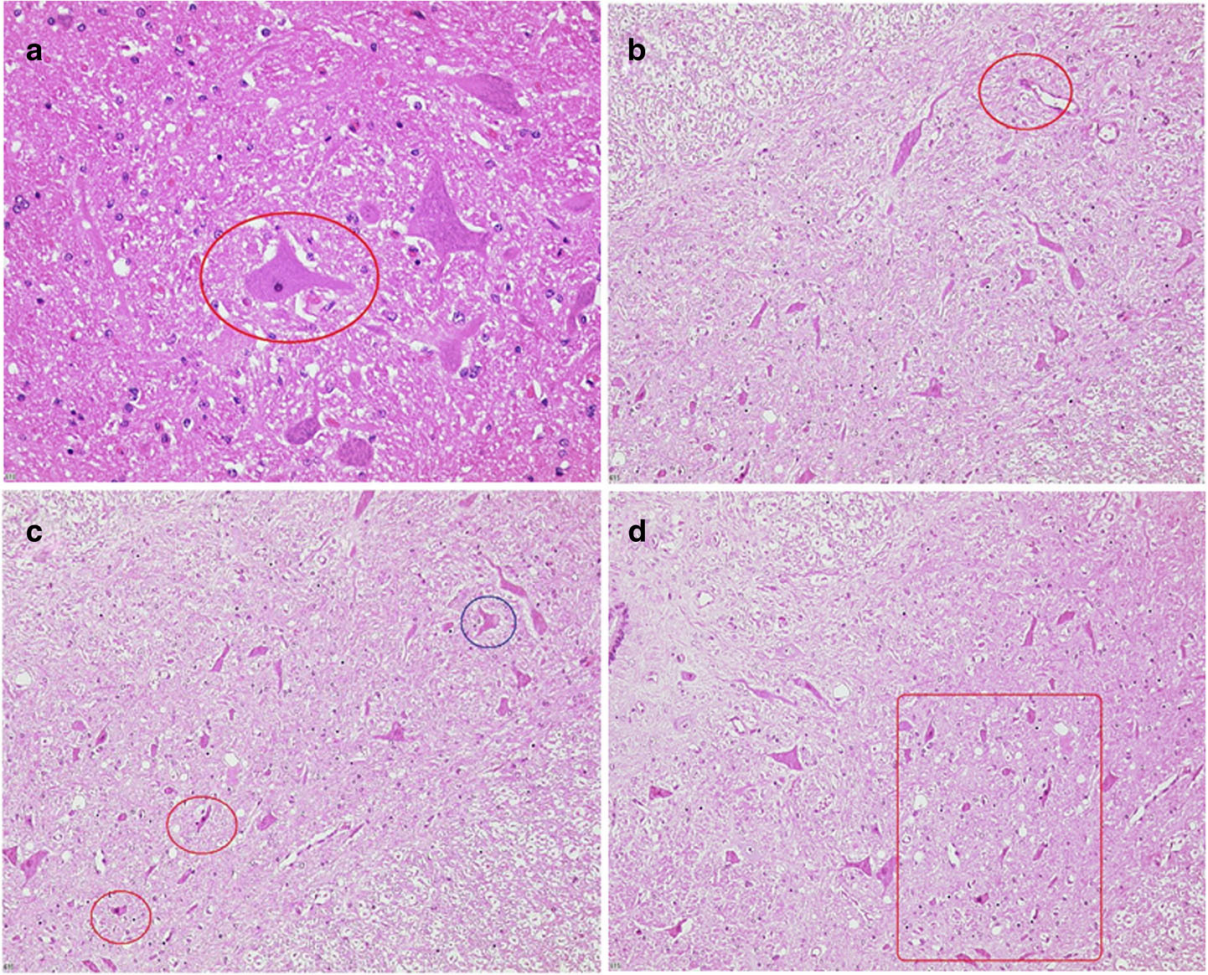
Histology of the spinal cord of familial hypercholesterolemia Bretoncelles Meishan (FMB) pig. **a** Red frame marks swollen motor neuron with pyknotic nucleus indicating neuronal damage. **b** Red frames mark endothelial damage. **c** Red frames mark ischemic neurons with eosinophilic cytoplasm and darkened, shrunken nuclei. Blue frames indicate the formation of a vacuole within a neuron. **d** Red frames indicate hypoxic damage with eosinophilic gangliocyte necrosis

**Fig. 3:**
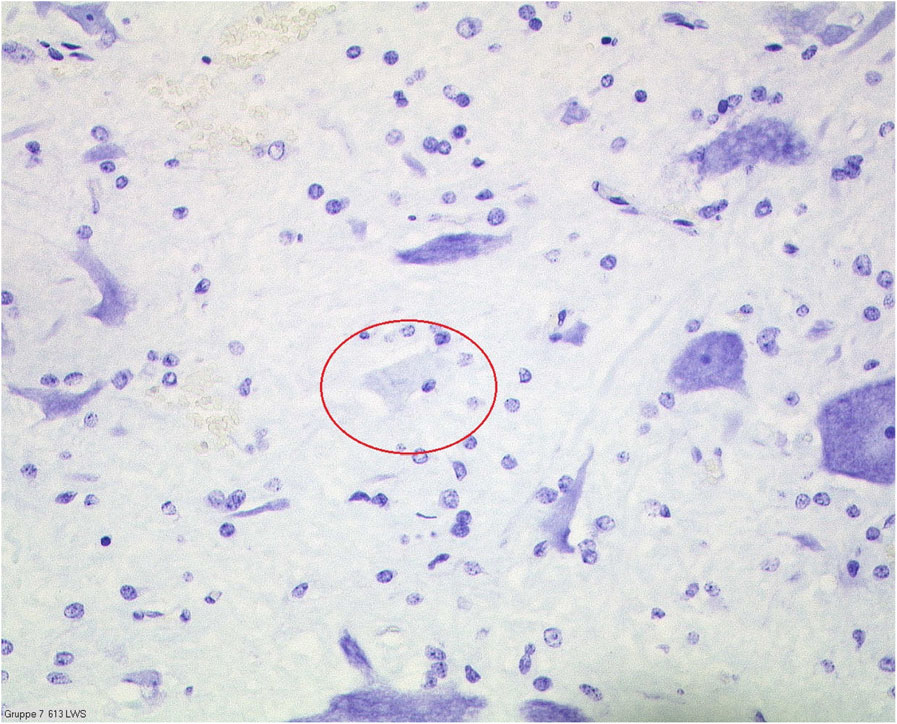
Histology of the spinal cord of familial hypercholesterolemia Bretoncelles Meishan (FMB) pig. Nissl staining of an ischemic infarction in the gray matter of an FBM pig on atherogenic diet after I/R injury. The red frame marks a necrotic neuron

In preliminary experiments, it was shown that an increase in the clamping time resulted in an increase in the neuronal damage (data not shown). A clamping time of 45 min caused a damage of neurons in the thoracic and lumbar spinal cord (mean proportion of damaged neurons, 16.4% and 16.2%, respectively). Treatment with sodium sulfide did not reduce the damage of neurons. The mean percentage of damaged neurons in the anterior horn of the thoracic and lumbar spinal cord was 21.1% and 23.9%, respectively. The slight increase in the sodium sulfide group might be a result of the animal with the large active infarction (see above). There was no significant difference between the vehicle and the sodium sulfide group. Typical histological examples are demonstrated in Figs. 2 and 3.

### Motor evoked potentials (MEPs)

MEPs of the spinal cord were used to measure the extent of the damage of the spinal cord after ischemic damage. In the study groups, two animals of the vehicle group and one animal of the sodium sulfide group showed small but measurable MEPs at 2 and 4 h after balloon deflating. The function also got lost after 8 h of reperfusion.

### Neurologic function

The clinical evaluation of the neurological function was evaluated at different time points. The reference at the upper extremities was always 7 points (3 points for normal reaction to claw clamping and 4 additional points for spontaneous movements). Furthermore, each animal showed maximal reflexes of the hind legs before balloon occlusion. In the sodium chloride group, only one animal showed link motions (2 points) at each time point during reperfusion. All other animals in both groups lost muscular reaction.

## Discussion

The present study tested the hypothesis if a (pre-)treatment with sodium sulfide (Na_2_S) would reduce neuronal damage in a clinically relevant porcine model of thoracic aortic balloon occlusion-induced spinal cord I/R injury. However, treatment with intravenous Na_2_S had no protective effect on I/R injury of the spinal cord.

In this study, swine were investigated because of their striking anatomical and physiological similarity to humans [31]. Furthermore, most studies focusing on pathophysiology utilize young animals, without any cardiovascular disease background, even though these animals are clearly not reflective of the pathophysiology of our patient population. However, the existence of atherosclerotic plaques in the vasculature represents an important independent risk factor for abdominal aortic aneurysms [32]. Therefore, we used a recently described porcine model of ubiquitous human-like atherosclerosis that is closer to the clinical situation for the therapy of aortic aneurysm [11, 12]. These FBM pigs are a cross-bread of Rapacz farm pigs with smaller Chinese Meishan and French Bretoncelles strains. They are homologous for the R84 low-density lipoprotein (LDL) receptor mutation and feature human-like thin-cap fibroatheromas (TCFA) at a young age after an atherogenic diet as previously described [11, 12]. They also have altered gene expression, like a downregulated erythropoietin receptor (EPO-R) expression, a lower systemic endothelial NO (eNO) production, but higher renal eNO levels when compared to other pig strains [12]. The downregulated EPO-R may be related to inflammation and oxidative stress and is a putative mechanism of EPO resistance in patients with heart and kidney failure [33, 34]. These circumstances might have contributed to the result that there was no attenuation of the I/R injury during Na_2_S infusion, especially since multiple studies have noted that the therapeutic effect of H_2_S donation requires the presence of an intact eNO synthase system [35–38].

The duration of aortic occlusion was based on previous experiments and the clinical situation of thoracic aortic cross-clamping in open surgical repair. In a pig model, this ischemia had resulted in neuronal injury (5–15% of all motor neurons) in the spinal cord at 4 h of reperfusion [23]. In addition, 45 min of aortic occlusion prevented both the large spinal cord infarction over several segments reported in pigs after a clamping period of 60 min or longer [24] and the fairly mild tissue damage observed after only 30 min of clamping [26]. In our pigs with a pre-existing ubiquitous atherosclerosis, 45 min of ischemia and 8 h of reperfusion had resulted in the same extent of neuronal damage. Furthermore, half of the non-treated animals developed an infarct at both levels of the spinal cord and only one animal recovered partial neuronal function with regain of MEPs (Table 2) and link motions (Table 3) at each time point after reperfusion. The restriction in regaining neuronal function was also observed in a previous study using a pig model with the same balloon occlusion time [30].

**Table 2:** Motor evoked potentials (MEPs) during the experiment

Group	MP1	MP2	MP3	MP4	MP5
Occlusion 30 min	2108 (100)	384 (18.2)	1351 (64.1)	656 (31.1)	495 (23.5)
Occlusion 40 min	841 (100)	0 (0)	0 (0)	0 (0)	74 (8.8)
NaCl (45 min)	2245 (100)	144 (6.4)	318 (14.2)	267 (11.9)	64 (2.9)
Na_2_S (45 min)	1148 (100)	23 (2)	25 (2.2)	10 (0.9)	6 (0.5)

All data are mean in microvolt and percentage of residual MEP compared to baseline (in brackets). Sodium chloride (NaCl), *n* = 4. Sodium sulfide (Na_2_S) *n* = 4. The data of the two groups with 30 min (*n* = 3) and 40 min (*n* = 2) occlusion time were obtained in preliminary experiments as described above. *MP* measuring point

**Table 3:** Clinical evaluation of hind motor function during the experiment. *MP* measuring point

Group	MP1	MP2	MP3	MP4	MP5
Occlusion 30 min	3 + 4 (2 × [3 + 4] + 1 × 3)	1 (1 × 1 + 1 × 2)	2 (1 × 2 + 1 × 1 + 1 × 0)	3 + 4 (1 × [3 + 4] + 2 × 0)	3 + 4 (1 × [3 + 4] + 1 × 1 + 1 × 0)
Occlusion 40 min	3 + 4 (2 × [3 + 4])	0 (2 × 0)	0 (2 × 0)	3 (1 × 3 + 1 × 0)	2 (1 × 2 + 1 × 0)
NaCl (45 min)	3 + 4 (4 × [3 + 4])	2 (3 × 0 + 1 × 2)	2 (3 × 0 + 1 × 2)	2 (3 × 0 + 1 × 2)	2 (3 × 0 + 1 × 2)
Na_2_S (45 min)	3 + 4 (4 × [3 + 4])	0 (4 × 0)	0 (4 × 0)	0 (4 × 0)	0 (4 × 0)

All animals showed normal function before aortic balloon occlusion. During reperfusion, only one animal in the sodium chloride group (*n* = 4) showed joint movement whereas all other pigs in the sodium sulfide (*n* = 4) group lost hind motor function. The data of the two groups with 30 min (*n* = 3) and 40 min (*n* = 2) occlusion time were obtained in preliminary experiments as described above. See text for details on the scoring system

This pig strain spontaneously develops hypothermia during anesthesia [12]. Core temperature was not externally influenced since hypothermia is an independent protective measurement possibly interfering with the effects of hydrogen sulfide [39, 40] and normothermia was not restored in order to avoid any pro-inflammatory and/or pro-apoptotic effects [41, 42].

The protective effects of compounds that release H_2_S endogenously on I/R injury are controversial. In porcine models of IR injury, Na_2_S exerted protective effects, such as maintaining mitochondrial function, neutralizing ROS and anti-inflammatory capacities, and reducing apoptosis especially in hearts and kidneys [5]. Administration of exogenous therapeutic Na_2_S prior to the onset of reperfusion after acute myocardial ischemia reduced the apoptotic response to I/R injury in a pig model [13]. In addition, its anti-inflammatory properties improved myocardial function and coronary microvascular reactivity by reducing infarct size [14, 15]. Ex vivo experiments of porcine kidneys, which were subjected to 25 min of warm ischemia and 18 h of cold storage under sulfide treatment, showed an improvement of renal function associated with a reduction of oxidative stress [43]. During porcine aortic occlusion-induced kidney I/R injury, pretreatment with Na_2_S attenuated tissue injury and organ dysfunction as a result of reduced inflammation and oxidative and nitrosative stress [27]. Administration of H_2_S in a large animal model of severe hemorrhagic shock resulted in a significant decrease in resuscitative requirements, decreased metabolic acidosis, and less end-organ histologic injury compared with standard resuscitation. On the other hand, it did not induce profound metabolic suppression as seen in rodents and appears to have alternative mechanisms of action in large animals, such as altered gene expression for hypoxia-inducible factor 1α (HIF 1α) and vascular endothelial growth factor receptor (VEGFr) [44]. In contrast, the present study showed that pretreatment with Na_2_S had no protective effect on the neuronal damage after aortic balloon occlusion in pigs. It could be speculated that the neuroprotection of sodium sulfide is dose-dependent. Current evidence seems to suggest that the presence of H_2_S in the ischemic brain may either be deleterious or protective depending on its concentration, deleterious when high and protective when low [45]. Deleterious actions include adversely influenced mitochondrial function, such as inhibited oxidative phosphorylation, as well as *N*-methyl-d-aspartate (NMDA) receptor and calcium-mediated cell death. H_2_S acts protective via its anti-inflammatory, anti-oxidative, and anti-apoptotic capacities [45]. Sulfide levels in our study were not measured because on the one hand sulfide interacts with numerous blood components (e.g., hemoglobin, iron, and dissolved oxygen) [46, 47]. For example, ex vivo spiking blood with defined amounts of sulfide does not allow for its quantitative recovery, so that sulfide administration in vivo may not necessarily yield detectable concentration changes unless toxic doses are used [48, 49]. In addition, it interferes with different reagents during chemical analysis, making it hard to measure accurate values. And a third point to be considered is that measured levels do not reflect the effect of the applied sulfide on the targeted cells or tissues. Different cells have different capacities to trap or oxidize sulfide. Especially cells with a high amount of metallo compounds or high expression of sulfide quinone reductase (e.g., hepatocytes or colonocytes) are immune against toxic sulfide levels whereas other cell types (e.g., cardiomyocytes or neurons) are more vulnerable [46, 47].

### Limitations of the study

It could be argued that we did not achieve sufficiently high sulfide levels in the spinal cord tissue. After administration of 3 mg/kg NaSH in rats challenged with traumatic brain injury, a significant rise of sulfide levels in brain tissue could be measured by Jiang et al. [50], suggesting that the comparable amounts in our experiment also yielded sufficient tissue sulfide levels. Nevertheless, since we did not measure tissue sulfide concentrations, we cannot exclude insufficient adsorption into the spinal cord due to different pharmacokinetics.

A balloon occlusion time of 45 min has probably been too long for the integrity of the spinal cord, mediating an irreversible I/R injury. In surgical settings, a maximum of 30 min for the cross-clamping is needed to prevent spinal cord injury. Another major limitation of this study was the low sample size of the experimental animals. However, since there was no improvement of the neurological damage after sodium sulfide application, the experiments were stopped for ethical reasons according to the 3Rs of animal research [51].

## Conclusion

In summary, in this porcine model of aortic occlusion-induced I/R injury, we demonstrated that pretreatment and continuous infusion with sodium sulfide did not protect spinal cord tissue from neuronal damage. Further studies are warranted to investigate the possible protective effects of different doses or other ways of application (e.g., inhalative), or other carriers of sulfide as sodium (e.g., chelates) on experimental models with pre-existing arteriosclerosis.

## Abbreviations

CVP: Central venous pressure
eNO: Endothelial nitrogen oxide
EPO-R: Erythropoietin receptor
FBM: Familial hypercholesteremia Bretoncelles Meishan
FiO_2_: Fraction of inspiratory oxygen
H_2_S: Hydrogen sulfide
HE: Hematoxylin and eosin
HIF: Hypoxia-inducible factor
I/R: Ischemia/reperfusion
IQR: Interquartile range
IU: International unit
LDL: Low-density lipoprotein
MAP: Mean arterial pressure
MEP: Motor evoked potential
MOF: Multi-organ failure
MP: Measuring point
NaOH: Sodium hydroxide
NaSH: Sodium hydrogen sulfide
NMDA: *N*-Methyl-d-aspartate
PEEP: Positive end-expiratory pressure
ROS: Reactive oxygen species
TCFA: Thin-cap fibroatheroma
VEGFr: Vascular endothelial growth factor receptor
